# Long-term evaluation of the prognosis of super hydrophilic surface treated CA implants: a retrospective clinical study

**DOI:** 10.1186/s12903-022-02142-0

**Published:** 2022-03-29

**Authors:** Min-Joong Kim, Il-hyung Kim, Na-Hee Chang, Young-Kyun Kim

**Affiliations:** 1 Department of Oral and Maxillofacial Surgery, Section of Dentistry, Seoul National University Bundang Hospital, 82 Gumi-ro 173beon-gil, Bundang-gu, Seongnam, 13620 Korea; 2 Department of Oral and Maxillofacial Surgery, Armed Forces Capital Dental Hospital, Armed Forces Medical Command, Seongnam, Korea; 3 Department of Biomedical Research Institute, Seoul National University Bundang Hospital, Seongnam, Korea; 4 School of Dentistry and Dental Research Institute, Seoul National University, Seoul, Korea

**Keywords:** Dental implants, Dental implantation, Endosseous, Dental materials, Osseointegration

## Abstract

**Background:**

This study was performed to evaluate the long-term clinical efficacy of the CA implants (Osstem Implant, Busan, Korea), calcium-modified surfaced treated implants on acid-etched surfaces sandblasted with alumina.

**Methods:**

From January 2013 to December 2015, 258 implants of 120 patients placed between 2013 and 2015 were retrospectively studied. Using medical records and periapical radiographs, sex, age, location, fixture width and length of placed implants, presence or absence of bone graft, types of bone substitutes and membrane used for bone grafting, primary and secondary stability, initial and delayed complications, and marginal bone loss were investigated. The success rate and survival rate of the implants in each group were analyzed retrospectively based on the criteria suggested by Albrektsson et al.

**Results:**

Between 2013 and 2015, with a follow-up longer than 5 years, 258 implants with an average diameter of 4.63 mm (3.5–5.5 mm) and an average length of 9.94 mm (7.0–13.0 mm) were placed in a total of 120 patients (61 males and 59 females) with a mean age of 63.7 years for an average of 62 months of observation period. The survival rate was 97.3%, the success rate was 94.2%, and the average final marginal bone loss was 0.074 mm.

**Conclusion:**

The CA implants manufactured with the improved surface treatment method exhibited a survival rate of 97.3% and a success rate of 94.2% over an average observation period of 62 months. The implants were not affected by most factors and had very high survival and success rates over a long period of observation. In particular, the stability of the implant was excellent, with no cases of failed implants in delayed placement after bone grafting and a healing period.

## Background

Today's implant fixtures are superior in many ways compared to the conventional ones. The earliest implants were mechanically polished flat surfaces, which were followed by more stable rough-surface implants. Therefore, the next issue was how best to produce a rough surface. The basis of most implant surface treatments currently in use is the Resorbable Blasted Media method, which increases fixation between bone and implant immediately after placement by increasing the surface area by spraying coated particles to create surface roughness. Many studies have been conducted on additional effective surface treatments. The Sandblasted, Large grit, and Acid-etched (SLA) method were developed as a mix of the blasting and acid-etched methods. In the SLA method, large particles (250–500 μm) are sprayed onto the surface to create areas of macro-roughness and micro-roughness, and then the roughness of the surface is maximized through acid corrosion (HCl/H_2_SO_4_) [1, 2]. The positive long-term prognosis of implants with SLA surfaces has been shown in several studies [3–8]. SA implants (Osstem Implant, Busan, Korea) were developed using SLA method, sandblasting and acid-etching using alumina. CA implants (Osstem Implant, Busan, Korea) are the same as conventional SA implants on surface topography but are stored in CaCl_2_ solution. Through this additional storage process, CA implants were developed to prevent carbon adsorption on the titanium surface of the implant, to improve wettability by increasing the surface activity, and to improve bone reaction performance at the initial stage of osseointegration due to increased protein adhesion to the surface [9].

Lee et al. reported that the survival rate within 1 year of early loading at 2 months after one-stage CA implant placement of a single maxillary molar was 96.4% [10]. In addition, Choi et al. performed follow-up observations of marginal bone resorption for 1 year after implantation in 107 cases of immediate posterior CA implantation. They found excellent marginal bone preservation of 0.09 mm at 3 months, 0.05 mm at 6 months, and 0.03 mm at 1 year [11].

However, most reports related to CA implants have been based on relatively short-term treatment results, and there is little information on long-term treatment prognosis. This likely is due to the relatively recent development and use of CA implants. Therefore, this study was conducted to investigate the treatment results and clinical applicability of calcium-modified SLA surfaces through a long-term retrospective study. In addition, since we authors have published a previous retrospective study on SA implants [8], the present study is meaningful as a secondary follow up.

## Methods

This retrospective clinical study was conducted after obtaining approval from the Bioethics Review Committee of Seoul National University Bundang Hospital (IRB No: B-2006-618-105), and was performed in accordance with the declaration of Helsinki. In order to analyze the cases of Osstem's CA surface implant placed at the section of dentistry at Seoul National University Bundang Hospital for more than 5 years as a focus target, 258 implants placed in a partially edentulous area that had been extracted for at least 6 weeks between 2013 and 2015 were the subjects of the study.

Using medical records and periapical radiographs, sex, age, location, width and length of the implants, presence or absence of bone graft, types of bone particles and membranes used during bone grafting, primary and secondary stability, early and delayed complications, and marginal bone loss were investigated. Patients with major risk factors such as radiation therapy, bisphosphonate use, or uncontrolled diabetes were excluded from the study.

Early complications were defined as infections or wound deterioration, necrosis, failure of initial fixation, early exposure of the fixture, mobility of the fixture upon hand torque, nerve damage, invasion into the roots of adjacent teeth after placement of the implant. These comprised complications that occurred before the implant procedure was complete. Delayed complications, on the other hand, referred to gingivitis, bone resorption around the implant, fracture of the implant fixture or prosthesis, and mobility of the fixture that occurred after normal functioning of the implant prosthesis.

Implant Stability Quotient (ISQ) measured by an Osstell Mentor (Osstell, Gӧteborg, Sweden) was used to determine the stability of the implant. ISQ was measured immediately after implant placement and was used to determine primary stability, while ISQ measured at the time of impression taking was a measure of secondary stability.

**Fig. 1:**
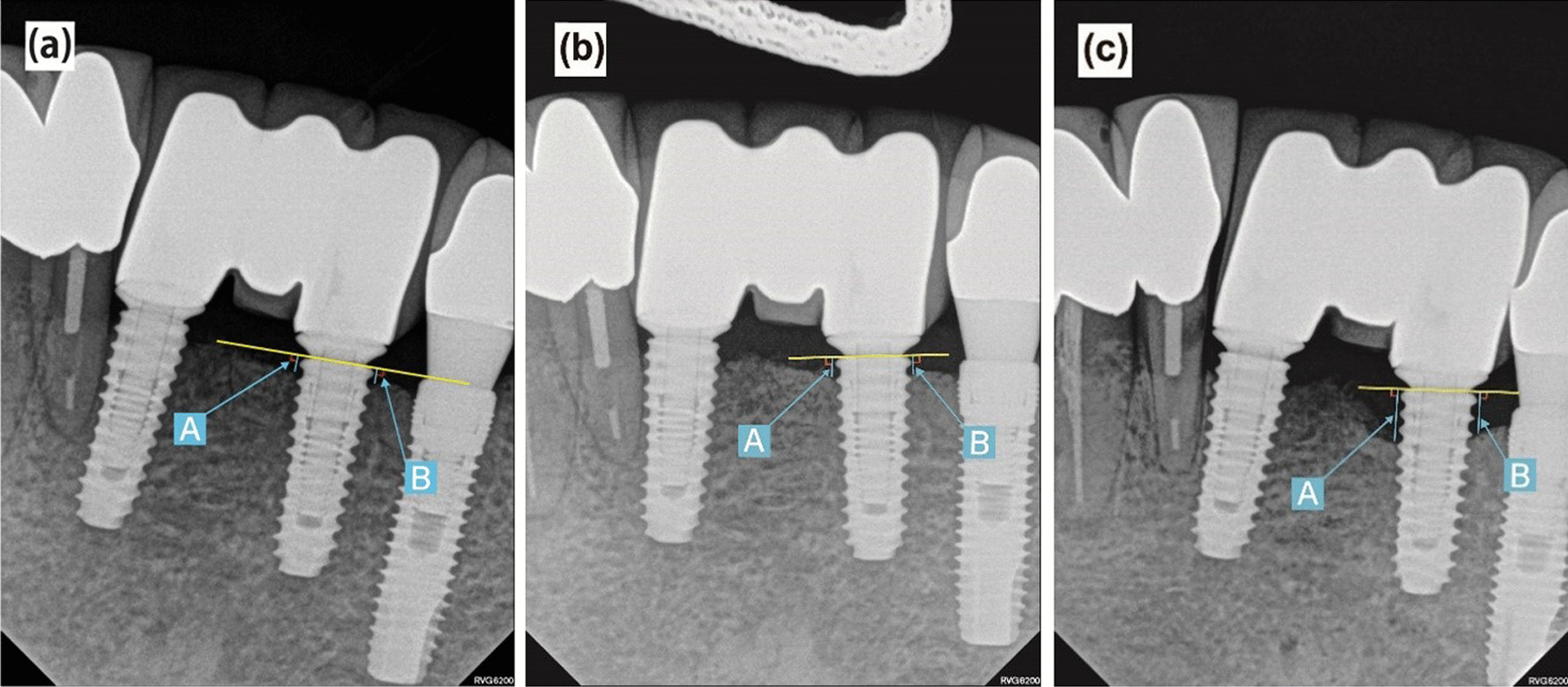
Landmarks of radiographic measurements. **a** Immediately after the prosthesis was installed; **b** Periapical view at 1-year after prosthetic loading; **c** Final periapical radiograph; **A** Shoulder to mesial contact point (mm); **B** Shoulder to distal contact point (mm); The average of A and B was the amount of marginal bone loss

The amount of marginal bone resorption was evaluated by comparing periapical radiographs with those from the time of implantation. Radiographs were produced with the paralleling cone technique using OC100 CR (Instrumentarium Imaging, Tuusula, Finland) and Heliodent DS (Sirona, Bensheim, Germany) radiation machines with INFINITT PACS 3.0 (INFINITT Healthcare Co., Ltd. Seoul, Korea) software. Radiological linear distances from each implant shoulder to the bone at the most proximal end of the implant and to the implant contact point were measured in the mesial and distal planes, and the average value was set as the marginal bone resorption amount (Fig. 1). At this time, to consider the magnification, the average alveolar bone absorption in the mesial and distal surfaces of the implants was calculated as the relative ratio of length of the implant fixture placed and he length of the fixture in the periapical view. The radiologic evaluation was performed twice by one oral and maxillofacial surgeon. Intraclass correlation coefficient was 0.81, indicating excellent reliability.

The criteria for implant success were set according to the following the criteria suggested by Albrektsson [11]: (1) No movement of the implant, (2) no radiolucent lesion around the implant, (3) no symptoms such as pain, discomfort, or infection, and (4) bone loss less than 0.2 mm every year after the first year of prosthesis function and of less than 1.5 mm during functional loading.

The criterion of implant success was set to a state of normal functioning without symptoms such as mobility of the fixture or patients' discomfort requiring explantation. The time point at which the complication of the implant occurred was set as the end date of implant success, and the survival date of the implant was the time point at which the implant was removed.

The survival rate, success rate, and complications of the implant according to factors of bone graft status, sex, implant length, and width were tested using IBM SPSS Statistics Ver. 25.0 (SPSS Inc., Chicago, IL, USA). Statistical analysis was performed through the Chi-square test, Fisher's exact test, and multiple regression analysis, which tested the significance of the association of the variables at a statistical significance level of 95%.

## Results

A total of 258 implants placed between January 2013 and December 2015 in 120 (61 males, 59 females) had an average observation period of 62.0 months in patients with an average age of 63.7 ± 11.17 years. The 258 implants were placed 33, 69, and 156 in the anterior, premolar, and molar regions, respectively, with 182 implants in the maxilla and 76 implants in the mandible.

The average primary stability was 70.57 ± 12.55, average secondary stability was 78.10 ± 8.00, survival rate was 97.3% (251/258), success rate was 94.2% (243/258), and marginal bone loss (mm) was 0.074 ± 0.26.

Of the total implants, 77 were placed without bone graft, 154 were placed simultaneous to bone graft, and 27 were placed at an average of 5.34 months after bone grafting. For implantation without bone graft (set as Group A), the average primary stability was 70.59 ± 12.57, and the average secondary stability was 78.85 ± 8.05. The survival rate was 98.7% (76/77), the success rate was 94.8% (73/77), and the average marginal bone resorption (mm) was 0.073 ± 0.26. In implantation with bone graft at the same time (set as Group B), the average primary stability was 70.57 ± 12.55, and the average secondary stability was 78.06 ± 8.0. The survival rate was 96.1% (148/154), the success rate was 92.9% (143/154), and the average marginal bone resorption (mm) was 0.074 ± 0.26. In delayed placement after bone graft (set as Group C), the average primary stability was 70.56 ± 13.20, and the average secondary stability was 77.94 ± 7.50. The survival rate was 100%, the success rate was 100%, and the marginal bone resorption (mm) was 0.000 (Table 1).

**Table 1:** Survival rates, success rates, marginal bone loss, and stability of the implants

Group	No. of implants	Survival rate (%)	Success rate (%)	Marginal bone loss (mm)	Stability (ISQ)	
					Primary	Secondary
Total	258	97.3	94.2	0.074 ± 0.26	70.57 ± 12.55	78.10 ± 8.00
A	77	98.7	94.8	0.073 ± 0.26	70.59 ± 12.57	78.85 ± 8.05
B	154	96.1	92.9	0.074 ± 0.26	70.57 ± 12.55	78.06 ± 8.00
C	27	100	100	0.000	70.56 ± 13.20	77.94 ± 7.50

Among the bone grafts used in a total of 181 cases in Groups B and C with bone graft, guided bone regeneration was used in 72, sinus elevation in 62, ridge augmentation in 21, extraction socket preservation in 3, and bone guided regeneration and maxillary sinus grafting were used in 23 cases. At the final follow-up of 258 implants, the types of prostheses were 146 cases with multiple fixed restorations, 104 cases with single fixed restorations, 3 cases with over dentures, 3 cases with two-unit bridges, and 2 cases of failure before implant restoration.

1. Implant stability

The average primary stability of the implants was 70.57 ± 12.55, and the average secondary stability was 78.10 ± 8.00. Of a total of 258 implants, 52 had a primary stability less than 60, and 3 implants had a secondary stability less than 60 (Table 2). The difference in implant stability among Groups A, B, and C, which had been followed for more than 5 years, was not significant (P > 0.05).

2. Complications

**Table 2:** Primary and secondary stability (ISQ) of the implants

Implant stability	ISQ		No
	Mean ± SD	Values	
Primary	70.57 ± 12.55	≥ 60	206
		< 60	52
Secondary	78.10 ± 8.00	≥ 60	255
		< 60	3

Complications occurred in 15 of 258 implants (5.8%), 2 with early complications and 13 with delayed complications. In 2 cases with initial complications, both failed, and in 13 cases with delayed complications, 5 cases with peri-implantitis failed. The types of initial complications were 1 case of osseointegration failure and 1 case of root involvement. The types of delayed complications were 8 cases of peri-implantitis and 5 cases of prosthetic complications (1 case of screw loosening, 2 cases of screw fracture, and 2 cases of food impaction). There was no statistically significant difference in the number of complications among Groups A, B, and C over more than 5 years of follow-up (P > 0.05).

3. Marginal bone loss

The average amount of marginal bone loss (mm) was 0.074 ± 0.26, and the average amount of bone loss at the time of one year after functioning was 0.056 ± 0.24. In a total of 258 cases, 248 (96.1%) experienced bone loss less than 1 mm within 1 year after implantation of the prosthesis, 8 (3.1%) of 1 mm or more but less than 2 mm, and 2 cases (0.8%) of implants that failed to survive due to initial complications before prosthesis placement. The difference in the amount of marginal bone resorption among Groups A, B, and C with follow-up over 5 years was not significant (P > 0.05).

4. Implant treatment outcomes according to diameter

A total of 7 implants dropped out, and 4 that were 5.0 mm in diameter and 3 that were 4.5 mm in diameter for each implant were excluded during function. Of the 15 implants that did not meet the success criteria, 10 were 5.0 mm in diameter, 4 were 4.5 mm in diameter, and one was 4.0 mm in diameter. The order of the average amount of marginal bone resorption by the diameter of implants at the most recent visit was 5.0 mm, followed by 4.0, 3.5, and 4.5 mm, and no marginal bone resorption was observed in the 5.5-mm-diameter implants (Table 3). Survival rate, success rate, and the amount of marginal bone resorption according to implant diameter were not statistically significant (P > 0.05).

5. Implant treatment outcomes according to length

**Table 3:** Survival rate, success rate, and marginal bone loss according to fixture diameter

Diameter (mm)	No. of implants			Survivor rate (%)		Success rate (%)	Marginal bone loss (mm)	
	Installed	Survived	Succeeded				1 year after placement	Most recent follow up
3.5	22	22	22	100	100		0.045	0.067
4.0	34	34	34	100	97.1		0.054	0.070
4.5	56	53	52	94.6	92.9		0.047	0.065
5.0	145	141	135	97.2	93.1		0.056	0.074
5.5	1	1	1	100	100		0.000	0.000

The seven implants that were eliminated numbered 5 at 10.0 mm and 1 each at 8.5 mm and 13.0 mm. The number of implants that did not satisfy the 5 success criteria comprised 10 at 10.0 mm, 2 at 8.5 mm, 2 at 11.5 mm, and 1 at 13 mm. The average amount of limbic bone resorption at the recent visit was highest in the 8.0 mm length implants, followed by those of 10.0, 11.5, 8.5 mm, and no marginal bone resorption was observed at 7.0, 10.5, and 13 mm (Table 4). There was no statistically significant difference in survival rate, success rate, and the amount of marginal bone resorption according to implant length (P > 0.05).

**Table 4:** Survival rate, success rate, and marginal bone loss according to fixture length

Length (mm)	No. of implants			Survivor rate (%)	Success rate (%)	Marginal bone loss (mm)	
	Installed	Survived	Succeeded			1 year after placement	Most recent follow up
7.0	1	1	1	100	100	0.000	0.000
8.0	1	1	1	100	100	0.200	0.250
8.5	56	55	54	98.2	96.4	0.047	0.064
10.0	158	153	148	96.8	93.7	0.056	0.074
10.5	1	1	1	100	100	0.000	0.000
11.5	34	34	32	100	94.1	0.048	0.068
13.0	7	6	6	85.7	85.7	0.000	0.000

## Discussion

According to the results of this study, no statistically significant differences were found among Groups A, B, and C in any of the factors regarding stability of the implant, complications, amount of marginal bone resorption, and diameter and length of the implant. These findings can be interpreted as excellent CA implant stability regardless of surgical modalities such as bone grafting, simultaneous implant placement with bone grafting, and delayed placement of implants after bone grafting.

Beginning in January 2013, only 7 of the 258 implants failed over an average of 5 years. Among the seven implants, two with initial complications were presumed to be caused by failure of initial osseointegration and combined apex lesions due to root invasion of adjacent teeth. The implants that failed to survive were immediately removed, and after a certain period of bone healing, implants were placed again through the submerged implantation method and have been well maintained without complications until now.

Sussman et al. reported that the prognoses of adjacent teeth and implant failures can be very poor due to traumatic damage to adjacent teeth caused by implant fixtures. He argued that if a patient complains of clinical symptoms due to traumatic damage to the adjacent tooth after implant placement or if periapical pathology is observed, the implant should be removed, and endodontic treatment for the tooth should be performed. In addition, it was argued that in situations where potential endodontic lesions of adjacent teeth might adversely affect implant osseointegration due to apex lesions after surgery, endodontic treatment of adjacent teeth should be performed prior to implant placement [13].

However, Yoon et al. reported that even if the implant fixture damages the roots of adjacent teeth, no harmful symptoms (for example, positive reactions with percussion test, tooth mobility, pulp necrosis) occur. If osseointegration of the implant is achieved, continuous use through follow-up is possible. They explained that no harmful symptoms occurred even when the implant fixture invaded the teeth, and reported that the reason was that it did not damage the cementum of the tooth root and thus did not cause pulpal damage [14]. In addition, Yi et al. reported that even if the surrounding natural teeth were damaged during implant placement, immediate extraction of adjacent teeth is not necessary, and that a conservative approach is sufficient to predict the prognosis of adjacent teeth. Osseointegration success rate and survival rate of the implant were high [15]. Although this study was conducted before digital dental implant technology was widespread, so all implantation was performed using conventional methods, one complication, in which the fixture invaded the adjacent root, is considered a problem that can be avoided if guided implantology and 3D-planning were used.

The remaining 5 of 7 implants that failed to survive all showed delayed complications, with peri-implantitis as the largest cause. As soon as symptoms of peri-implant mucositis or peri-implantitis were observed, peri-implant curettage, chlorhexidine solution irrigation, and minocycline ointment application were performed under local anesthesia as the primary treatment methods. If symptoms were not alleviated, open flap debridement, cleaning using a titanium brush, tetracycline application, and detoxification treatment using laser were performed as a secondary treatment method. If the amount of bone defect was severe, additional bone grafting was performed. Removal and re-implantation of the 5 implants was planned after the symptoms of peri-implantitis continued to worsen after treatment, eventually resulting in mobility of the implant fixture with inflammation. However, of the 8 cases of peri-implantitis, except for 5 of which failed to survive, the complications could be resolved through the above treatment. All prosthetic delayed complications, except peri-implantitis, such as screw loosening, screw fracture, food impaction, and occlusal disorders, were resolved through repair and remanufacture of the prosthesis.

Several studies have demonstrated the effectiveness of chlorhexidine and minocycline as non-surgical supportive treatment in peri-implant mucositis [16, 17]. However, if peri-implantitis develops beyond peri-implant mucositis and bone loss begins to progress, it is recommended to proceed with surgical treatment [18, 19].

Toma et al. reported that, when peri-implantitis occurs, curettage using a plastic curette, a method using an air-abrasive device, and a cleaning method using a titanium brush are very effective for removing inflammation. In addition, it was reported that the use of antibiotic therapy together with these physical methods could lead to more effective results [20]. In particular, John et al. reported that removing plaque using a titanium brush is more efficient than any other method using plastic or metal curettes [21]. In addition, in recent studies, physical methods such as curettage, titanium brushing, and implantoplasty have emerged as effective methods because they do not reduce the biocompatibility between the titanium surface of the implant fixture and osteoblasts [22].

In addition, when the prognosis of the implant is poor due to excessive marginal bone resorption, additional bone grafting around the implant fixture was performed as a reconstructive procedure. Bassi et al. reported that additional bone grafting at the end of long-term peri-implantitis management and treatment increased radiopacity around the implant and was able to effectively fill bone defects [23].

Two implants that did not survive due to osseointegration failure were considered early implant failures, and five implants that failed to survive due to peri-implantitis were considered delayed failures. Early implant failure typically is caused by disruption of the initial healing process due to formation of abnormal tissue between the surface of the implant and the bone due to infection, trauma during surgery, excessive load, or tissue necrosis. Delayed implant failure is attributed to a pathological phenomenon in which the biological equilibrium around the implant is disrupted due to inflammation around the prosthesis and implant fixture due to various factors [24–29].

In this study, implants with delayed implantation after a healing period from primary bone graft did not experience failure or showed a small amount of marginal bone resorption. Recently, the immediate implant placement method has received more attention than the delayed method to reduce patient discomfort and maintain soft-tissue morphology. However, in several papers comparing the long-term stability of implants, it has been argued that implants with delayed placement are more stable than immediately placed implants. Haas et al. reported that immediate implant placement was more time-efficient compared to delayed placement and could be a more aesthetic treatment option but did not show the same success rate as delayed placement [30]. In addition, Kunnekel et al. found that osseointegration in delayed implantation occurred much faster than did that of immediate implantation according to resonance frequency analysis [31].

## Conclusion

The TS III CA implant with the super hydrophilic surface treatment method had a survival rate of 97.3% and a success rate of 94.2% over an average observation period of 62 months. The stability of the implant was excellent, and there were no instances of failure in delayed placement.

## Data Availability

The data that support the findings of this study are available from the approval of administration of the corresponding author's affiliated institution but restrictions apply to the availability of these data, which were used under license for the current study, and so are not publicly available. Data are however available from the corresponding author upon reasonable request and with permission of the institution.
